# The Effect of Glycemic Status on P Wave Dispersion

**DOI:** 10.7759/cureus.58233

**Published:** 2024-04-14

**Authors:** Niranjan Ragavan, Rida Thavalam, Swathy Moorthy

**Affiliations:** 1 Internal Medicine, Sri Ramachandra Institute of Higher Education and Research, Chennai, IND

**Keywords:** atrial fibrilation, risk factors cardiovascular diseases, type 2 diabetes mellites, p wave dispersion, cardiovascular prevention

## Abstract

Background and aim

The growing number of people with diabetes mellitus (DM) across the world is a public health concern. The diabetes epidemic involves enormous health costs to the patients, their careers, and society at large. Cardiovascular diseases such as atrial fibrillation (AF) often develop in the diabetic population. An increase in the P wave dispersion (PWD) has been established as an independent risk factor for the occurrence of AF, hence the present study was conducted to establish a possible relationship between PWD and the glycemic status of the individual to predict the occurrence of AF ahead of clinical symptomology.

Methodology

A comparative cross-sectional study was conducted at a tertiary care hospital after obtaining approval from the institutional ethics committee and written consent of each study subject.

The main steps included the selection and categorization of the study population based on their glycemic status, collection of demographic data, performing ECGs calculating PWD using digital calipers, and recording the data systematically for evaluation.

Results

In this study, 234 patients with a mean age of 53.3 ± 13.1 years were studied, of which 121 (51.7%) were male and 113 (48.29%) were female. The 234 patients were divided into four groups based on their glycemic status - 74 uncontrolled DM patients (31.62%), 51 type 2 DM (T2DM) patients (21.78%), 56 prediabetes patients (23.93%), and 53 patients in the control group (22.64%; not a known case of diabetes with normal HbA1c and fasting blood sugar (FBS) levels). Minimal correlation was observed between FBS with PWD (r value 0.175) and age with PWD (r value 0.161), but statistical significance was observed only between age and PWD (p-value 0.014). The difference in means between the four different study groups was found to be not statistically significant (p-value- 0.104); hence, no intergroup variation was noted.

Conclusion

Advancing age and higher fasting blood sugars have shown minimal correlation with widening P-wave dispersion. With further studies involving larger populations, this can be a promising aid in identifying PWD as a probable early predictor of atrial arrhythmias among diabetic patients.

## Introduction

Diabetes mellitus (DM) is a lifestyle disorder that poses a significant risk for the occurrence of cardiovascular diseases (CVD). At a global scale, CVDs affect approximately 32.2% of all persons with T2DM [1]. A consistent global rise in the number of diabetic patients poses a major burden on public health, placing long-term and unsustainable demands on the patients and burdening the healthcare system. The latest estimates show a global prevalence of 537 million adults with diabetes in 2021, which is projected to rise to 783 million by 2045 [2]. India already has the maximum number of people estimated to be diagnosed with DM (approximately 73 million), which suggests every fifth individual with diabetes in the world is from India [3].

Cardiac disease that develops as a direct consequence of DM in patients with type 1 DM (T1DM) or type 2 DM (T2DM), known as diabetic heart disease, has characteristic molecular, structural, and functional changes in the myocardium [4]. Diabetes confers a 1.4- and 1.6-fold risk of CVD for men and women after adjusting for other associated conditions, respectively [5].

P wave dispersion (PWD) measures the heterogeneity of atrial refractoriness and is defined as the difference between the maximum P wave (P max) and minimum P wave (P min) durations on the standard 12-lead electrocardiography (ECG). An increase in PWD is suggestive of intra-atrial and inter-atrial non-uniform conduction [6]. 

The exact mechanism of PWD prolongation in DM patients is not well known. Structural and electrophysiological changes in the atrial myocardium caused by diabetes have been proposed to be contributing factors. Long-standing hyperglycemia leads to changes in the chemical composition of cell membrane protein structure [6]. Moreover, extracellular protein deposition and interstitial fibrosis of the myocardium can lead to heterogeneity in atrial conduction velocity and atrial refractoriness in diabetic patients, which can cause prolongation of PWD. Hence, it has been indicated that PWD can aid in diagnosing patients with a high risk of developing arrhythmias [7].

Atrial fibrillation (AF) is the most commonly and frequently diagnosed sustained tachyarrhythmia. The asymptomatic nature of this arrhythmia substantiates the need for a target-driven approach to prevention. The electrocardiogram (ECG) has become an important tool for this purpose. Several studies have demonstrated that DM and poor glycemic control reflected by glycated hemoglobin (HbA1c) levels are independently associated with new-onset AF [8].

Prediabetes (PD) is diagnosed with impaired fasting glucose (IFG) or impaired glucose tolerance (IGT) [9]. Every stage of glucose abnormalities is associated with a greater risk of developing cardiovascular disease (CVD), which makes it essential to screen and diagnose them early. Development of vascular damage starts early, even before the diagnosis of diabetes is established in certain patients. Hence, the glucose-metabolic perturbation can often stay sub-clinical until the time of occurrence of a cardiovascular event [10].

Meta-analyses and observational studies have shown that patients with uncontrolled diabetes (UDM) are at an increased risk for CVD and mortality compared to patients with controlled diabetes [11]. This study, therefore, aims to measure and compare the degree of variation of PWD among the different study subgroups with T2DM and the correlation between PWD with age and glycemic parameters. This would aid in evaluating the individual and relative risk for developing atrial fibrillation and prove to be a target-driven comprehensive correction of the modifiable risk factors.

## Materials and methods

A comparative cross-sectional study was conducted for a period of two months between August to October 2022, in a tertiary care center. The study was conducted after obtaining prior approval from the Institutional Ethics Committee (study number CSP/21/AUG/97/416) with recruitment of study subjects done after obtaining their written informed consent.

The study population was grouped into four categories: group A with 56 prediabetes patients (23.93%), group B with 51 T2DM patients (21.78%), group C with 74 uncontrolled DM patients (31.62%), and group D with 53 patients in the control group (22.64%) -Patients who are not a known case of DM/ CVD were the controls. All patients were over the age of 18 years and were included in the study based on the American Diabetes Association definition (ADA) [12] as prediabetes (HbA1c of 5.7% to 6.4% with impaired fasting glucose of 100-125 mg/dl), type 2 diabetes mellitus (HbA1c of ≥6.5% and fasting plasma glucose of 126 mg/dl or higher) and uncontrolled diabetes mellitus (HbA1c of >7%). Patients with other comorbidities such as structural heart defects, history of myocardial infarction (MI), ischemic changes in the ECG, previous history of AF, permanent pacemaker implantation, patients with a history of antiarrhythmic drug usage, and patients who had unclear P waves in more than four leads on a baseline 12-lead ECG were excluded from the study.

The basic demographic, clinical, and laboratory data collected were entered into a pre-structured proforma and were later analyzed. The glycated hemoglobin (HbA1c) levels were obtained using high-performance liquid chromatography (HPLC) with a Tosoh G8 analyzer (Tosoh, Tokyo, Japan). Blood sugar levels were measured with Beckman AU 5800 & AU680 Automated clinical chemistry analyzer (Beckman Coulter, Brea, California). The standard 12-lead surface electrocardiography was recorded and used to measure the PWD using a recorder at 25 mm/s paper speed and 10 mm/mV standardization (MAC 600 with Marquette® 12SL™ analytical tools; GE HealthCare, Chicago, Illinois). Recordings were done on the patient while relaxed, taking spontaneous breathing, and in the supine position.

Measurement of P waves

The beginning of the P wave is defined as the point of the first upward slope visible from the baseline for positive waveforms and as the point of the first downward slope from the baseline for negative waveforms. The return to the baseline was considered as the end of the P wave. Biphasic P waves were measured at the time of final return to the baseline.

P maximum (P max) was defined as the longest atrial conduction time as measured on any of the 12 leads, and the shortest time was defined as P minimum (P min) and P wave dispersion (PWD = P max - P min) as the difference between P max and P min. ECGs were scanned using an Adobe scanner (Adobe, Mountain View, California) and measured digitally at a standard magnification of 300x. Zhart digital calipers (0.01 mm precision), with a linear capacitive measuring system, were used to measure the P waves. The values were methodically recorded in seconds (s) and calculated further by a single trained observer.

Statistical analysis

Data analysis was done with the SPSS statistics software for Windows (version 23.0; Armonk, New York). The patient's age and gender were expressed as number (%), the glycemic values were presented as mean +/- standard deviation, and difference in means between the four study groups was tested using ANOVA. Pearson's correlation was used to assess the relationship between glycemic parameters and PWD. A probability value of <0.05 was considered statistically significant.

## Results

The study enrolled a total of 234 patients, with the mean age in the study group being 53.3 ± 13.1 years. The lowest age of the enrolled patients was 22 years and the eldest patient enrolled was 86 years old. The current study had a near-equal sex distribution of male and female patients with 121 males (48.29%) and 113 females (51.7%).

The glycemic status of the study participants was assessed by random blood sugar (RBS), fasting blood sugar (FBS), postprandial blood sugar (PPBS), and glycated hemoglobin (HbA1c) levels. The baseline characteristics of the study subjects are compiled in Table 1. Table 2 shows the baseline characteristics and PWD of the study participants across the different subgroups. The prediabetes group had a slightly higher aged population compared to the rest of the groups.

**Table 1:** Baseline characteristics of the study population (n=234) RBS - random blood sugar; FBS - fasting blood sugar; PPBS - post prandial blood sugar

Parameter	Mean ± standard deviation
Age (years)	53.35 ± 13.179
RBS (mg/dl)	154.75 ± 82.987
FBS (mg/dl)	145.17 ± 80.740
PPBS (mg/dl)	233.15 ± 138.541
HbA1c (%)	7.39 ± 2.379

**Table 2:** Baseline characteristics and P wave dispersion of the study groups RBS - random blood sugar; FBS - fasting blood sugar; PPBS - post prandial blood sugar; P max - P maximum; P min - P minimum; PWD - P wave dispersion; C - control; PD - prediabetes; T2DM - type 2 diabetes mellitus; UDM - uncontrolled diabetes mellitus

Parameter	C (n=53)	PD (n=56)	T2DM (n=51)	UDM (n=74)
Age (years)	46.42 ± 14.25	56.82 ± 13.98	53.86 ± 10.66	55.35 ± 11.66
RBS (mg/dl)	105 ± 30.93	111.02 ± 34.43	147.55 ± 46.352	219.64 ± 100.71
FBS (mg/dl)	101.85 ± 32.75	121.44 ± 55.28	115.04 ± 23.27	202.62 ±104.52
PPBS (mg/dl)	127.67 ± 51.702	169.92 ± 94.73	159.57 ± 50.984	348.48 ± 138.26
HbA1c (%)	3.98 ±2.32	5.673 ± 1.39	6.592 ±0.955	9.99 ± 2.35
P max (Seconds)	0.417 ± 0.135	0.543 ± 0.49	0.045 ± 0.126	0.052 ± 0.012
P min (Seconds)	0.177 ± 0.0065	0.188 ± 0.006	0.018 ± 0.006	0.019 ± 0.005
PWD (Seconds)	0.0253 ± 0.0089	0.0354 ± 0.049	0.0262 ± 0.008	0.0324 ± 0.0097

Table 3 shows Pearson's correlation between p-wave dispersion and age, FBS, and HbA1c of the study group. Minimal correlation was observed between FBS with PWD (r value 0.175) and age with PWD (r value 0.161), but statistical significance was only observed between age and PWD (p-value 0.014). The difference in means between the four different study groups was tested by ANOVA and was found to be not statistically significant (p-value 0.104). 

**Table 3:** Pearson's correlation of P wave dispersion with demographics and glycemic parameters PWD - P wave dispersion; FBS - fasting blood sugar; HbA1c - glycated hemoglobin *p-value less than 0.05

Parameter	Correlation value with PWD	p-value
Age	0.161	0.014*
FBS	0.175	0.116
HbA1c	0.040	0.556

**Figure 1:**
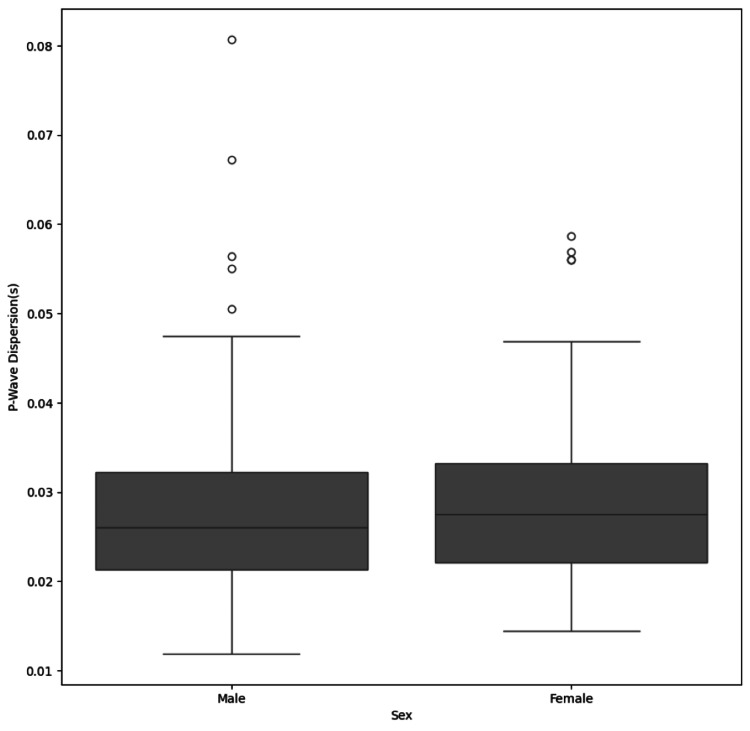
Box plot distribution between sex and P wave dispersion

**Figure 2:**
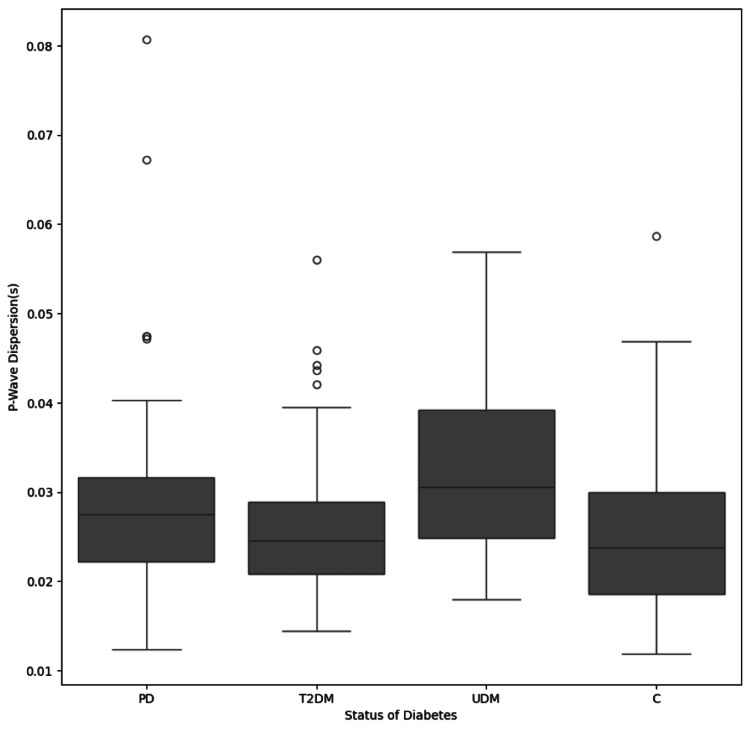
Box plot distribution between study groups and P wave dispersion PD - prediabetes; T2DM - type 2 diabetes mellitus; UDM - uncontrolled diabetes mellitus; C - control group

**Figure 3:**
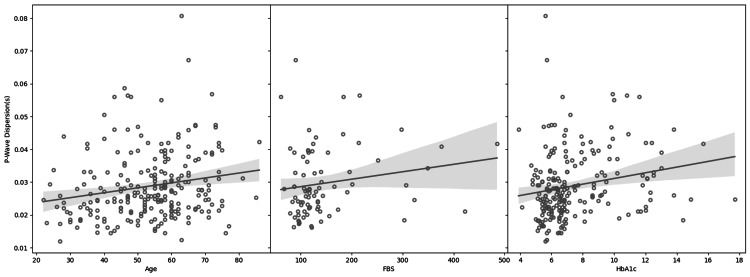
Correlation between age and PWD, FBS and PWD, and HbA1c and PWD PWD - P wave dispersion; FBS - fasting blood sugar; HbA1c - glycated hemoglobin

Figure 1 shows the distribution between sex and p-wave dispersion. Figure 2 shows the box plot distribution between the different subgroups and P wave dispersion. Scatter plots for correlation between P wave dispersion and age, FBS, and HbA1c are shown in Figure 3.

## Discussion

DM has been established as an independent risk factor contributing to changes in cardiac structure and function. Diastolic dysfunction is regarded as one of the first signs of functional myocardial abnormalities and can be detected in almost 75% of asymptomatic DM patients [13].

The pathophysiological mechanisms for the development of AF among diabetics are still a concept under constant review. Some of the implicated mechanisms are autonomic, electromechanical, structural changes, oxidative stress, glycemic fluctuations, and connexin remodeling. DM-related atrial fibrosis can result in prolonged atrial activation time and cycle length, along with a reduction of local atrial electrogram voltages, thus contributing to the development of arrhythmias [8].

A 1.4- to 2.1-fold increase in the occurrence of AF is noted among the diabetic population compared to the normal population [14]. Intraatrial and interatrial conduction disorders cause a multitude of electrophysiologic and electromechanical abnormalities, which result in a higher risk of AF. The interatrial electromechanical delay (EMD) and concomitant inhomogeneous propagation of sinus impulses make the atrial muscle prone to developing fibrillation [15].

Dilaveris et al. introduced the parameter P wave dispersion (PWD) in 1998 as a marker of nonuniform, inhomogeneous, anisotropic atrial conduction, and its presence poses a risk for the development of atrial fibrillation (AF) [16]. This concept has been around in clinical use for more than 20 years, but the factors that are giving rise to it have not yet been accurately defined. PWD is considered by most researchers to be the result of inhomogeneous conduction of the atrial electrical impulse, but its origin could also be explained by a vectorial projection [17]. PWD measurement is done based on assuming that surface ECG represents partial regional changes in the areas of myocardial activation. However, the determination of PWD solely by the heterogeneity of atrial conduction or through other factors remains unclear [18]. 

In their follow-up study, Dilaveris et al. compared the parameters between 60 patients with a history of paroxysmal lone AF and 40 healthy controls. PWD was significantly higher among patients with paroxysmal lone AF (p-value <0.0001). They suggested a cut-off value of 40ms as significant PWD, proving to have a sensitivity of 83%, a specificity of 85%, and a positive predictive accuracy of 89% for the same, with a relative risk of recurrence of AF being 2.4 times more for a PWD value >40 ms, during a 12 month follow up [19].

Karabag et al. conducted a study to determine the prevalence of PWD in pre-diabetic patients. The results showed no correlation was found between PWD and HbA1c levels (r=19; p>0.05), but a positive correlation was observed between PWD and FBG (r=0.32; p<0.01) [20]. In our study, PD patients had no significant correlation with PWD. However, there was a trend toward significance, which could be partly due to the smaller sample size and the variations in methodology, as quoted in the literature [21].

Demir et al. assessed atrial electromechanical delay (EMD) and P wave dispersion in patients with type 2 diabetes mellitus. P max duration and PWD were found to be significantly higher in patients with T2DM [22]. However, no such significant correlation was observed in our study. Magnani et al. demonstrated that age had a significant positive association with all P wave parameters [23], which was observed even in our study. This can be attributed to the fact that the elasticity of myocardial fibrils gradually decreases with age, and fibrosis increases atrial refractoriness, which prolongs conductivity. This, when superimposed with the effects of DM, increases the proneness of arrhythmias.

Currently, PWD is calculated based on the absolute difference between the shortest and longest P waves from the surface ECG. Greater sensitivity for the detection of inhomogeneity of atrial activation can be achieved by using adjacent leads with shared vectorial orientation [24]. Hence, future studies should examine the heterogeneity of leads in findings.

Methodologies and data collection processes in studies measuring PWD differ significantly in applying automated versus manual measurements ECG scanned and digitized compared with ECG printed on a strip of paper, paper speed, and printed resolutions. In our study, we scanned the ECGs and measured the P waves manually using digital calipers under magnification. Dilaveris et al., in another study, pointed out that manual measurement of P waves in standard 12-lead ECGs, measured on a high-resolution screen, is more stable and reliable than methods that involve paper-printed ECGs, which are more conventional [25].

A possible limitation of this study is the lesser number of study participants in each group and its single-center nature. ECG tracing artifacts may influence even very precise measurements and also need to be taken into account. Also, the utility of adjacent leads with shared vectorial orientation was not attempted, which could have provided better sensitivity for inhomogeneity of atrial activation detection. In a study conducted on healthy individuals, P max, P min, and P wave dispersion traversed a broad range of values. The high variability of P wave parameters in healthy individuals and the overlapping results with those reported for patients with increased risk for atrial fibrillation might indicate the limited sensitivity and specificity of the technique [21]. The variation observed between studies probably arises from methodological issues, and therefore, there is a definite need for methodological standardization of PWD measurements.

## Conclusions

The study was designed to assess the predictive value of P wave dispersion for atrial fibrillation among the diabetic population. However, no significant difference in P wave dispersion among the varying spectrum of type 2 diabetes mellitus was seen. A minimal correlation was seen between PWD with FBS values and the age of the study population, but could not establish any statistical significance.

Expanding the study with a higher study population can probably aid in PWD being identified as an early predictor of AF among diabetic patients. Establishing novel cut-off values of PWD, in cases of AF, including its predictive value for risk assessment in patients with type 2 diabetes mellitus is important for early detection of the condition before the onset of clinical symptomology.
